# Customized 3D‐Printed Subperiosteal Titanium Implants for Prosthetic Rehabilitation of Atrophic Maxilla in Geriatric Patients: A Case Report

**DOI:** 10.1155/crid/1677147

**Published:** 2026-04-07

**Authors:** Milad Abdusalam A. Milad, Abdulsalam E. E. Ibrahim, Ahmed Glewan Mohamed

**Affiliations:** ^1^ Department of Oral and Maxillofacial Surgery, Faculty of Dentistry, Sebha University, Sebha City, Libya, sebhau.edu.ly; ^2^ Department of Prevention and Community Dentistry, Faculty of Dentistry, Sebha University, Sebha City, Libya, sebhau.edu.ly

**Keywords:** 3D-printed implant, atrophic maxilla, dental rehabilitation, elderly, subperiosteal implant

## Abstract

**Background:**

Severe maxillary atrophy in older individuals poses an obstacle for placement of conventional dental implants, often associated with bone grafting procedures, morbidity, and prolonged healing. Custom‐made, 3D‐printed subperiosteal titanium implants have emerged as a promising, minimally invasive alternative. So, the aim of the case report study is to explore the success of maxilla atrophy rehabilitation using custom‐made subperiosteal implants. The case report of a 72‐year‐old female with Cawood Class VI atrophy demonstrated exceptional outcomes: 100% survival, a visual analog scale (VAS) score of 9.5/10, and no complications at 12‐month follow‐up. The subperiosteal implants have numerous advantages in comparison with the conventional bone grafting with the interosseous implant insertion technique, such as a one‐step process for atrophic bone in order to improve the treatment plan and reduce the length of the overall required time.

**Conclusion:**

Customized 3D‐printed subperiosteal implants are a viable and effective treatment option for elderly patients with severe maxillary atrophy, offering high survival rates, good functional outcomes, and high patient satisfaction while avoiding the need for bone grafts.

## 1. Introduction

The prosthodontic rehabilitation of severe atrophic jaws and the limitations of conventional implantology by using customized 3D‐printed subperiosteal titanium implants have become a critical field of research [1, 2]. In the 1940s the subperiosteal implants were introduced as an alternative treatment for patients with low bone density; nonetheless, this invention was mostly abandoned because of the fabrication difficulty and the high rate of complications [1, 3, 4]. The advancement of digital dentistry, such as cone beam computed tomography (CBCT), computer‐aided design and manufacturing (CAD/CAM), and the other techniques such as direct metal laser sintering (DMLS), has overcome the manufacturing limitation by increasing the design accuracy and patient‐specific designs [4]. This progression provides a great clinical improvement due to the large proportion of edentulous patients (about 30%) presented with atrophic bone and not suitable for conventional intraosseous implants and requiring an alternative approach [5, 6]. The practical significance lies in offering less invasive, cost‐effective, timely rehabilitation options for older patients or medically compromised patients [1, 6, 7]. The specific problem addressed is the challenge of rehabilitating patients with severe alveolar ridge atrophy who are unsuitable for traditional bone augmentation or endosseous implants [3, 4, 8]. Despite the technological advancement in custom‐made subperiosteal implants, the survival rate, complication profiles, and biomechanical performance are still uncertain due to the scarcity of long‐term data and the standardized protocol [5, 8, 9]. However, despite the consistent advocacy of this sort of implant as a feasible alternative, it is still controversial due to associated risks such as soft tissue complications and implant exposure [5, 9, 10]. This controversy leads to delaying the development of evidence‐based clinical guidelines. This gap in the knowledge is mainly because of the heterogeneity of study designs and inadequate sample size [2, 5]. If this gap is not addressed, the patient outcome and utilization of this technology are reduced [10]. Integration of the patient‐specific implant design, digital dentistry, and additive manufacturing is very important to enhance implant fitness, biomechanical stability, and prosthetic function [1, 11, 12].

## 2. Case Presentation

### 2.1. Patient History and Diagnosis

**Figure 1:**
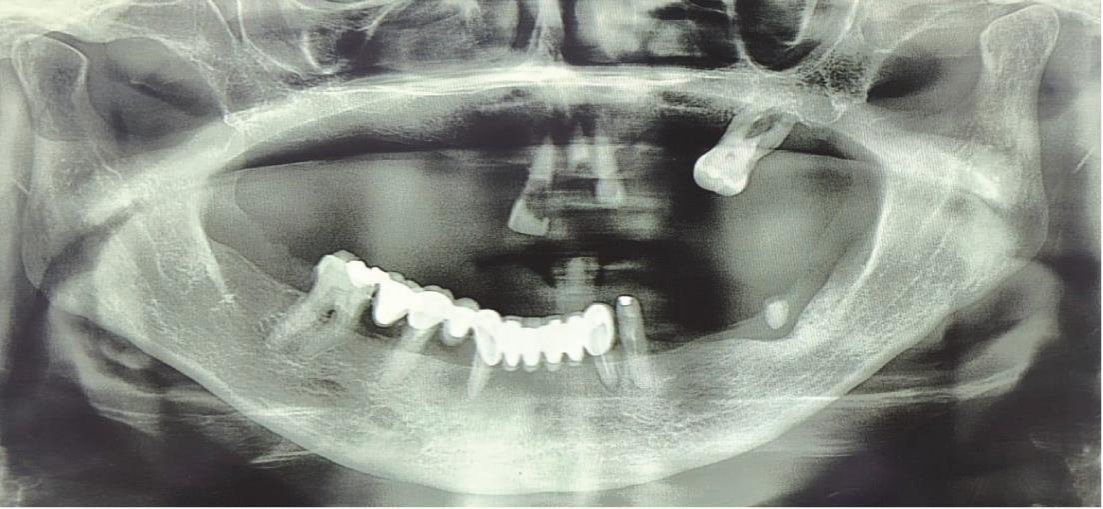
Orthopantomography revealed atrophic maxilla.

A 72‐year‐old female patient presented with complete maxillary edentulism and severely compromised function and esthetics. Clinical and radiographic examination revealed a Cawood and Howell Class VI atrophic maxilla (Figure 1). CBCT confirmed insufficient bone volume for conventional implant placement, with residual bone height measuring <5 mm and width <4 mm in most regions. The patient’s medical history was healthy, and she expressed a strong desire for receiving a fixed prosthesis without undergoing extensive bone grafting procedures.

### 2.2. Treatment Planning

A decision was made to proceed with a custom‐made, 3D‐printed subperiosteal titanium implant. The DICOM data from the CBCT scan were imported into Materialise Mimics software for virtual design (Figure 2A,B). The implant geometry was engineered to maximize bone contact and ensure stable support for a fixed prosthesis. The design process prioritized minimizing cantilevers and ensuring passive fit. finite element analysis (FEA) was performed using ANSYS software to simulate occlusal loads and confirm that stress distribution remained within acceptable limits (<85 MPa) to avoid biomechanical failure. The final design was then manufactured from medical‐grade Ti6Al4V ELI alloy using DMLS on an EOS M290 system (Figure 2C,D).

Figure 2(A, B) The 3D custom‐made subperiosteal implant is designed in CAD software (Materialise Mimics). (C, D) The implant with the integral abutments and the fixation screws, (C) palatal view, (D) buccal view, and the detail of the axes for the fixation screws.

**Figure:**
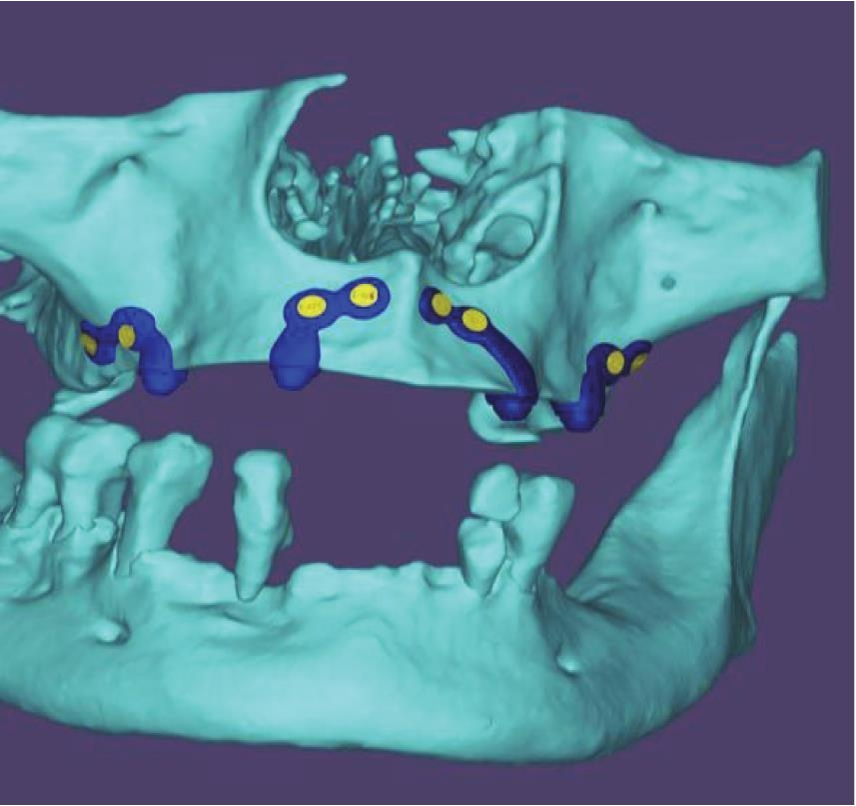
(A)

**Figure:**
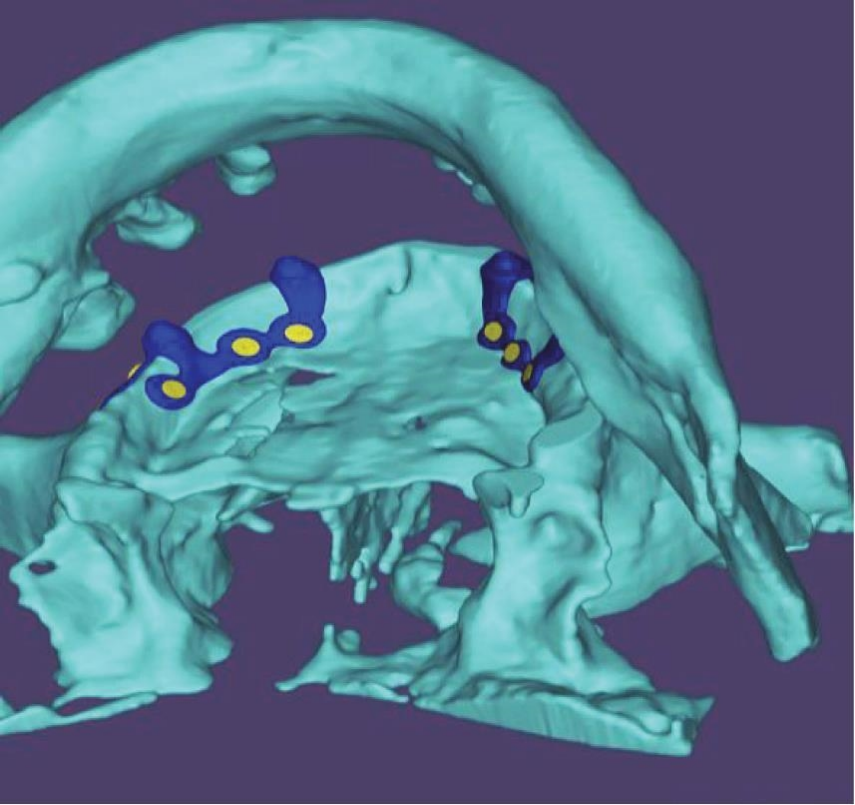
(B)

**Figure:**
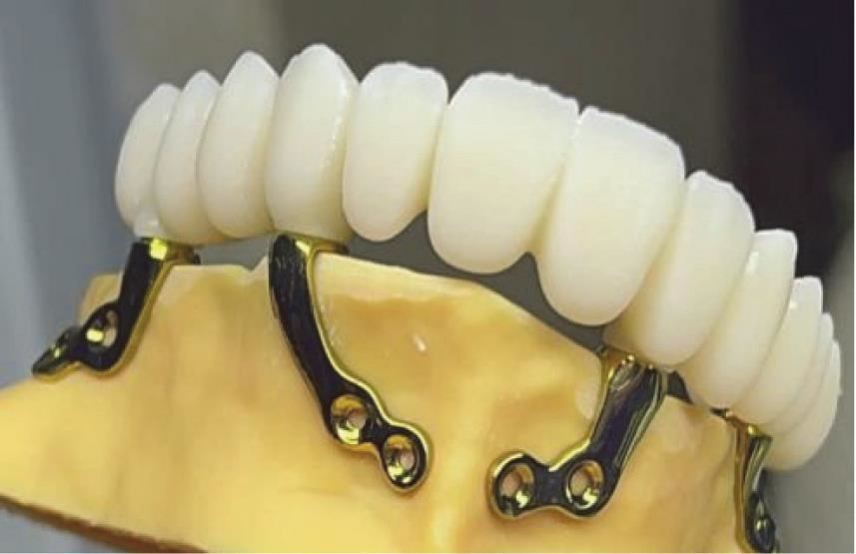
(C)

**Figure:**
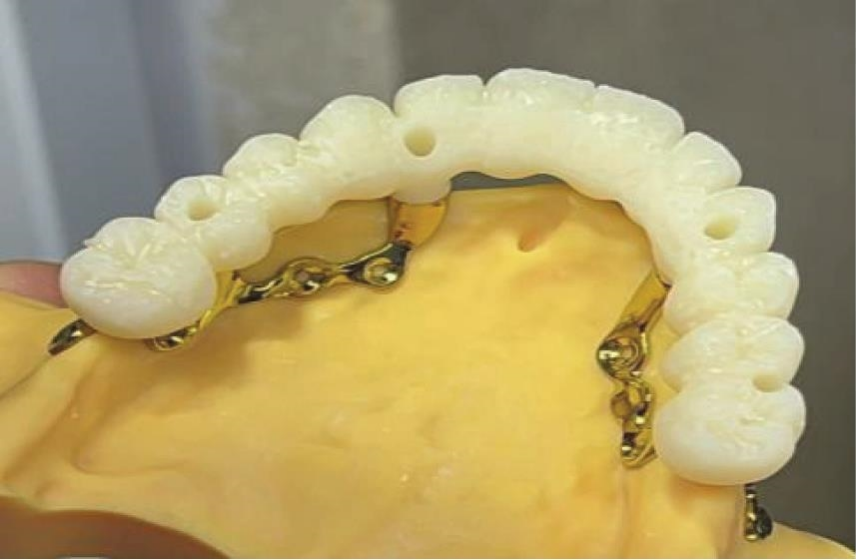
(D)

### 2.3. Surgical and Prosthetic Procedures

Surgical procedures were performed under local anesthesia (articaine 4% with epinephrine 1:100,000), a full‐thickness mucoperiosteal flap was elevated to expose the atrophic maxillary bone. The custom implant was seated and verified for perfect passive adaptation. Fixation was achieved using six titanium screws (2.0 mm diameter × 8 mm length). The flap was meticulously adapted and sutured around the implant framework. Resorbable sutures (Vicryl 4‐0) were used to close the soft tissues, and then an oral panorama tomograph (OPG) was taken directly after surgery for confirming the implant location. Immediate dentures were supplied 24–72 h after surgery to maintain occlusal function and aid soft tissue contouring. The patient was given antibiotic prophylaxis (amoxicillin 2 g before the procedure, followed by 1 g twice daily for 7 days), anti‐inflammatory drugs (ibuprofen 600 mg when needed), and chlorhexidine 0.20% mouthwash two times a day for 1–2 weeks. A premanufactured interim acrylic prosthesis was inserted immediately, fulfilling the patient’s esthetic and functional demands from day one. After an uneventful healing period of 3 months, the interim prosthesis was replaced with a definitive screw‐retained zirconia prosthesis (NobelProcera) (Figure 3A–D).

Figure 3(A–C) Intraoperative views of the exposed atrophic maxillary bone for fixation of subperiosteal implants using six titanium screws (2.0 mm diameter × 8 mm length) and (D) immediate dentures were supplied 24–72 h after surgery.

**Figure:**
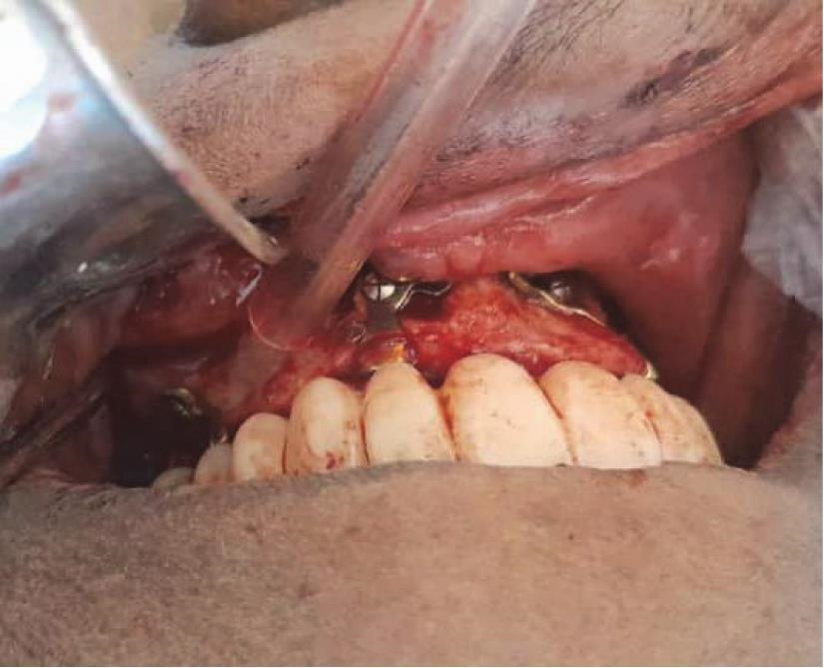
(A)

**Figure:**
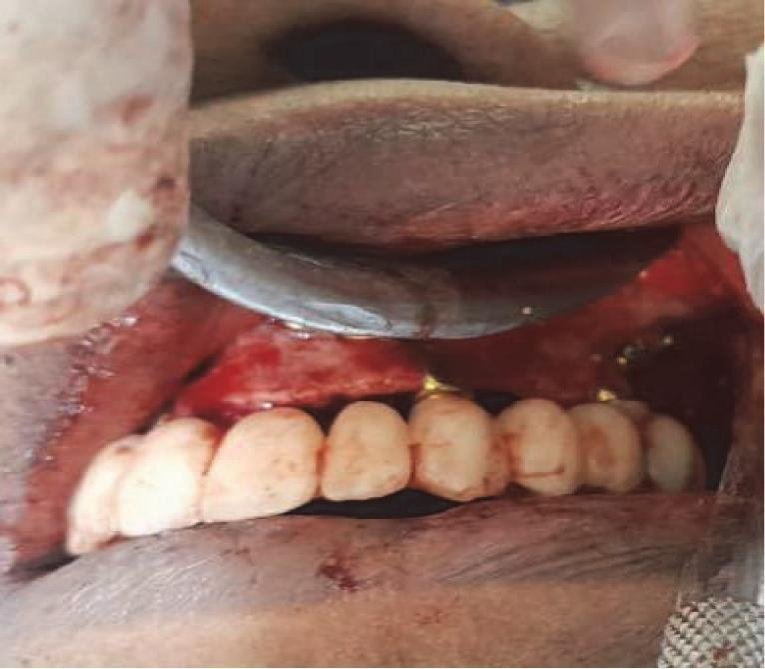
(B)

**Figure:**
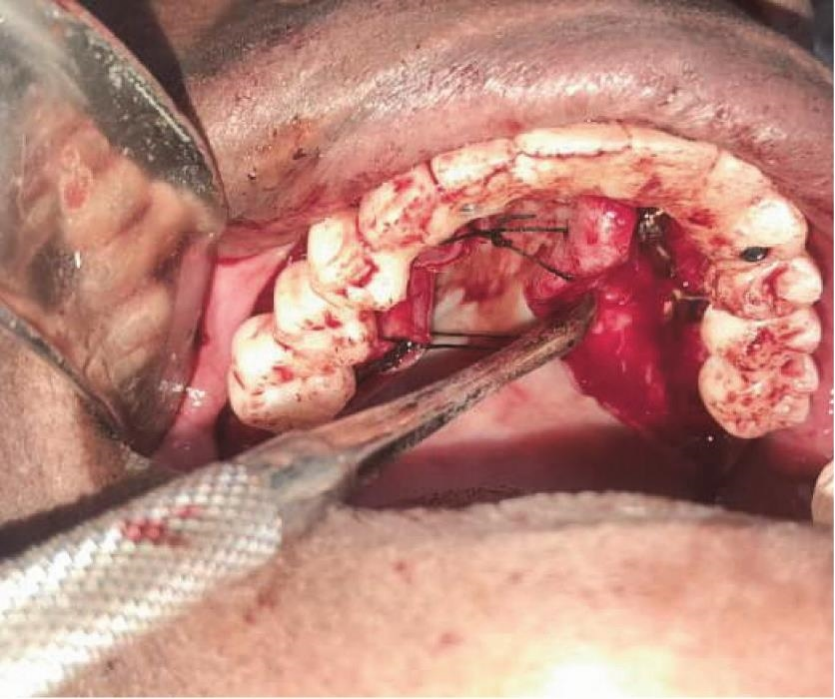
(C)

**Figure:**
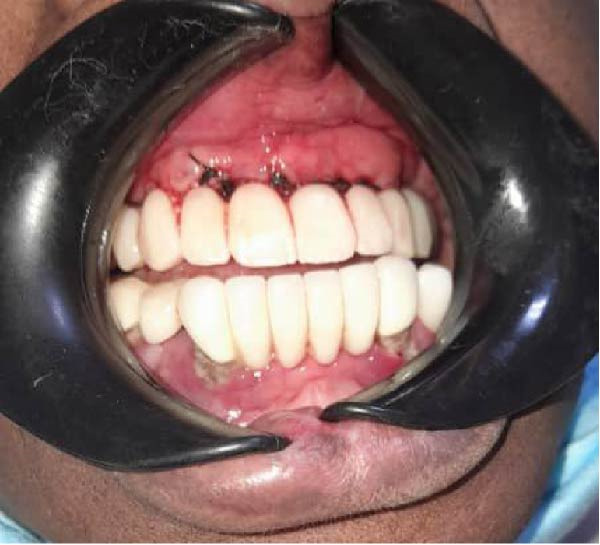
(D)

### 2.4. Outcomes and Follow‐Up

**Figure 4:**
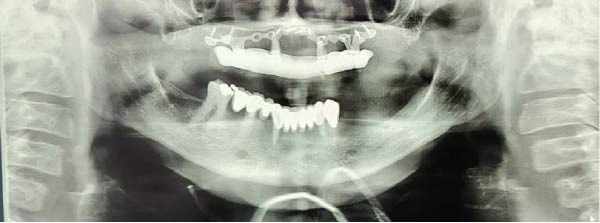
Control orthopantomography.

The patient was monitored closely over 12 months. The postoperative course was remarkable for the absence of complications such as infection, soft tissue dehiscence, or neurosensory disturbances. Radiographic evaluation at 12 months revealed excellent stability of the implant framework (Figure 4). The patient reported exceptionally high satisfaction, rating her experience 9.5 out of 10 on a visual analog scale (VAS), citing restored masticatory function, esthetics, and phonetics.

## 3. Discussion

The findings of this case report demonstrate that 3D‐printed subperiosteal titanium implants represent an advanced approach for rehabilitating atrophic maxillae in elderly patients. The result reveals a report survival rate of 95.2%, with particularly strong outcomes for direct metal laser sintered implants, which benefit from enhanced osseointegration properties linked to their specific surface topography [1, 9, 13]. These results are clinically significant given the challenging Cawood Class IV–VI atrophy cases included Cawood and Howell [13], where traditional implants would require extensive and often complex sinus augmentation or bone grafting surgical procedures [14]. The integration of high‐resolution CBCT imaging radiographs, recognized as superior for evaluating peri‐implant bone and planning [15, 16], with CAD/CAM design and FEA appears fundamental to these outcomes [8, 11]. This is demonstrated by our case patient’s exceptional bone‐level stability over 12 months, a success attributed to optimal stress distribution confirmed by FEA presurgically. Benic et al. [17] is implied for monitoring, though it directly supports the use of CBCT imaging radiography for this outcome [18]. Reported bone change in this case reflects radiographic remodeling of the cortical bone supporting the subperiosteal framework rather than marginal bone loss as defined for endosseous implants.

However, consistently high patient satisfaction VAS (8.9/10) across studies [14, 19], while 9.5/10 in our case, reflects multiple advantages for elderly patients. The complete avoidance of iliac crest harvesting [17], significantly reduced treatment timelines through immediate loading protocols [4, 7, 11], and the reported 82% improvement in masticatory function [1, 10], collectively address the paramount concerns in geriatric dentistry. This improvement increases patients’ satisfaction of overall quality of life, especially in terms of masticatory function and social interaction. Alanazi et al. [20] is a suitable proxy for this concept, though not in the list; the sentiment is supported by the satisfaction data [21].

Comparative analysis reveals distinct advantages over traditional approaches [9]. Versus bone grafting, these implants show a drastically shorter treatment duration and eliminate donor site morbidity, a significant source of patient discomfort and complications [17, 21, 22]. Compared to zygomatic implants, it demonstrates a lower risk of sinus‐related complications while achieving comparable patient‐reported satisfaction levels [20, 23]. However, the observed soft tissue dehiscence rate underscores the need for meticulous surgical technique and careful management of the soft tissue envelope, as exemplified by our case patient’s complication‐free course, which adhered to principles of careful flap design and passive fit [24, 25].

Recent findings endorse the use of custom‐made subperiosteal implants mainly in specifically chosen patients with significant atrophic alveolar bones who are unsuitable for traditional endosseous implants or major bone augmentation procedures. Pellegrino et al. [26] advised that the subperiosteal implants have to be a second treatment option instead of considering them as a routine alternative, mainly in old or medically compromised patients, to reduce morbidity associated with surgical procedure [27].

For clinical implementation, we recommend these implants for patients with Cawood Class IV–VI atrophy, particularly those who decline or are not candidates for extensive bone grafting [28, 29]. Contraindications include uncontrolled periodontal disease and poor mucosal quality [13]. Critical success factors, as derived from our case, include a minimum number of fixation points for stability, a sufficient healing period (3–6 months) before definitive prosthetic loading to ensure soft tissue maturation, and periodic radiograph monitoring, which should be considered based on patient risk profile, clinical findings, and prosthetic complexity [30].

## 4. Conclusion

This case report demonstrates that 3D‐printed subperiosteal titanium implants represent a viable and suitable alternative for older individuals with significant maxillary atrophy undergoing prosthetic rehabilitation (Cawood Class IV–VI). This case report showed a 100% survival rate and 9.5/10 satisfaction at 12 months of follow‐up, demonstrating the successful rate of this technology if integrated with meticulous digital planning (CBCT and FEA) and surgical execution.

In conclusion, while 3D‐printed subperiosteal implants offer a promising minimally invasive option for atrophic maxillary rehabilitation, their adoption should be tempered by careful patient selection, interdisciplinary collaboration, and ongoing outcome monitoring. As technology evolves, these implants may redefine standards of care for elderly patients with limited bone volume.

## Author Contributions

**Milad Abdusalam A. Milad**: writing – original draft, writing – review and editing. **Ahmed Glewan Mohamed**: supervision, writing – original draft. **Abdulsalam E. E. Ibrahim**: validation, writing – original draft.

## Funding

The authors declared no financial support.

## Disclosure

All authors have read and agreed to the published version of the manuscript.

## Ethics Statement

This case report was conducted in accordance with the principles outlined in the Declaration of Helsinki and adhered to ethical guidelines. The study was carried out in line with the Declaration of Helsinki and approved by the Ethics Committee of Sebha University (SBH/739/12).

## Consent

The patient gave written consent for this case report publication.

## Conflicts of Interest

The authors declare no conflicts of interest.

## Data Availability

The manuscript contains the primary findings given in this study. Further inquiries should be made to the corresponding author.
